# Phylogenetic Relationships and Evolutionary Patterns of the Order Collodaria (Radiolaria)

**DOI:** 10.1371/journal.pone.0035775

**Published:** 2012-05-02

**Authors:** Yoshiyuki Ishitani, Yurika Ujiié, Colomban de Vargas, Fabrice Not, Kozo Takahashi

**Affiliations:** 1 Japan Agency for Marine-Earth Science and Technology (JAMSTEC), Yokosuka, Kanagawa, Japan; 2 Center for Advanced Marine Core Research, Kochi University, Nankoku, Kochi, Japan; 3 UMR CNRS 7144 Evolution du Plancton et PaleOceans, Station Biologique, Roscoff, France; 4 Earth and Planetary Sciences, Department of Sciences, Kyushu University, Hakozaki, Higasi-ku, Fukuoka, Japan; J. Craig Venter Institute, United States of America

## Abstract

Collodaria are the only group of Radiolaria that has a colonial lifestyle. This group is potentially the most important plankton in the oligotrophic ocean because of its large biomass and the high primary productivity associated with the numerous symbionts inside a cell or colony. The evolution of Collodaria could thus be related to the changes in paleo-productivity that have affected organic carbon fixation in the oligotrophic ocean. However, the fossil record of Collodaria is insufficient to trace their abundance through geological time, because most collodarians do not have silicified shells. Recently, molecular phylogeny based on nuclear small sub-unit ribosomal DNA (SSU rDNA) confirmed Collodaria to be one of five orders of Radiolaria, though the relationship among collodarians is still unresolved because of inadequate taxonomic sampling. Our phylogenetic analysis has revealed four novel collodarian sequences, on the basis of which collodarians can be divided into four clades that correspond to taxonomic grouping at the family level: Thalassicollidae, Collozoidae, Collosphaeridae, and Collophidae. Comparison of the results of our phylogenetic analyses with the morphological characteristics of each collodarian family suggests that the first ancestral collodarians had a solitary lifestyle and left no silica deposits. The timing of events estimated from molecular divergence calculations indicates that naked collodarian lineages first appeared around 45.6 million years (Ma) ago, coincident with the diversification of diatoms in the pelagic oceans. Colonial collodarians appeared after the formation of the present ocean circulation system and the development of oligotrophic conditions in the equatorial Pacific (ca. 33.4 Ma ago). The divergence of colonial collodarians probably caused a shift in the efficiency of primary production during this period.

## Introduction

Radiolaria are classified in the Rhizaria super group together with Foraminifera, Endomyxa, and Filosa [1]. Radiolaria have characteristic cell structures that include axopodia, an internal endoplasm that contains the nucleus and major metabolic organelles, and an external ecoplasm that is separated from the endoplasm by a central capsule [2]. Photosynthetic symbionts are typically found in the ectoplasm. On the basis of a combination of morphological and molecular phylogenetic analyses, the Radiolaria have been assigned to five distinct orders: Spumellaria, Acantharia, Taxopodida, Nassellaria, and Collodaria [3].

Taxonomically, Collodaria have unique morphological and ecological features among Radiolaria, because this order includes species with colonial lifestyles and without silicification (i.e., naked). On the basis of these features, Collodaria have been classified into three families: Thalassicollidae, Collosphaeridae, and Collozoidae [2]. Only Thalassicollidae are characterized by solitary cell without a silica skeleton. The other two families have colonial lifestyles, and some of them have silica skeletons. Morphological characterization of the siliceous skeletons divides the Collozoidae and Collosphaeridae on the basis of whether the cells possess siliceous spines or an irregular latticed shell, respectively [2]. Moreover, taxonomical schemes [4]–[7] have been used as a basis for subdividing the family Collozoidae into three genera according to the morphological characteristics of the spines: *Collozoum* has no spines; *Rhaphidozoum* possesses simple spines; and *Sphaerozoum* has spines with a characteristic triangular shape (spicule) [4]. Radiolarian morphological taxonomy has thus been based on some of the characteristics of the siliceous skeletons (spines or shells) outside the endoplasm. The other shell-bearing radiolarian orders (Spumellaria, Nassellaria, and Acantharia) are generally classified on the basis of the structure of the inner shell, which is located inside the endoplasm [8]. Indeed, molecular phylogenetic assessment has shown the distinct lineages of Spumellaria to be congruent with their inner shell structure [9]. However, there is a lack of comparable taxonomic criteria for Collodaria because of the absence of the inner shell structure. Exterior morphological analysis has led to the inclusion of species of the collodarian genus *Collophidium* in the genus *Collozoum*, because both taxa consist of naked colonial cells. In contrast, an ultra-structural study (e.g., of the shape of the central capsule and nucleus) has shown that the characteristics of the nucleus and central capsule of *Collophidium* and *Collozoum* differ sufficiently to recommend re-establishment of the genus *Collophidium* [7]. Although some studies have tried to approach collodarian taxonomy on the basis of cell structure characteristics [10], [11], these studies have been examined in few taxa and are insufficient to classify all collodarians.

Instead, a molecular phylogenetic study has recently reported the phylogenetic relationship among collodarians [12]. The collodarian taxa form a robust monophyletic clade at the order level in the radiolarian phylogeny [3], as same as in the phylogeny including diverse group of eukaryotes [12]. However, the monophyletic clade of family Collosphaeridae has been nested in the multidivergent clade of family Collozoidae [12]. Moreover, the phylogenetic position of the genus *Collophidium* is unknown, because there has been only one taxonomic sampling.

Collodarians, which are a highly diverse order of Radiolaria in the oligotrophic tropical and subtropical oceans, are ecologically categorized as persistent obligatory acquired phototrophic marine protists bearing photosythetic endosymbionts [13]. The high rate of carbon fixation by photosynthetic endosymbionts supplies collodarians with nutrition. The fact that colonial collodarians (Collosphaeridae and Collozoidae) possess substantial numbers of symbionts (2×10^6^ cells in a large colony [14]) results in a high rate of primary production in a colony (e.g., 1400–41,000 ng carbohydrate colony^–1^ hour^–1^ [15]). Collodarians are able to survive in oligotrophic environments by exploiting this high potential for carbon fixation (e.g., carbohydrate in *Collosphaera huxleyi*: 91.16 µg [16]). Even in the solitary group (Thalassicollidae), collodarians have the potential to keep carbon reserves inside a cell (carbohydrate in *Thalassicolla nucleate*; 0.16 µg [16]) and achieve high primary production rates (10–64 ng carbohydrate hour^–1^ [16]). Both solitary and colonial collodarians tolerate oligotrophic conditions. Collodarians could thus contribute substantially to carbon fixation in the oligotrophic ocean. In the Gulf of Aden, for example, the amount of carbon fixed by collodarian endosymbionts is estimated to be three times the carbon fixed by free phytoplankton in the water column surrounding the colony [17], [18]. This high carbon fixation ability could affect biogeochemical cycles in oligotrophic waters. Collodarians have probably evolved specific adaptations that enable them to flourish in oligotrophic environments. However, the adaptive responses of collodarians to oligotrophic environments have not been evaluated on the basis of fossil evidence, because most collodarians (nearly all species of Thalassicollidae and Sphaerozoidae) have no siliceous structures. Molecular divergence time estimates, however, will improve our understanding of their ecological impact in the paleoceanographic history.

Here, we inferred the SSU rDNA phylogeny of Collodaria concerning four novel sequences. Our analysis revealed phylogenetic relationships among the collodarian families and led us to revise the taxonomic scheme and criteria for classification of Collodaria. Divergence time estimates of major collodarian lineages showed diversification to be well correlated with paleoceanographic events. In our discussion we have examined the co-evolution of collodarian lineages and the development of the oligotrophic oceans throughout the Cenozoic Era.

## Results

### SSU rDNA phylogeny

**Figure 1:**
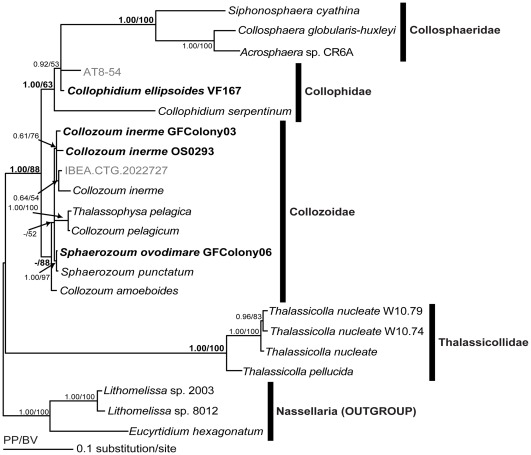
Bayesian phylogeny of the SSU rDNA sequences obtained from the collodarians. Four novel collodarian sequences and two environmental sequences (<3 µm) are shown in bold and light gray text, respectively. Family names are shown to the right of the black bars. Numbers on nodes indicates posterior probabilities of the Bayesian method and bootstrap values of the ML analysis. Scale bar at lower left shows 0.1 substitutions per site for the Bayesian analyses.

We confirmed that all 19 collodarian SSU rDNA sequences were associated with the single monophyletic clade of Collodaria in the radiolarian phylogeny (Fig. S1). The Bayesian phylogeny of these collodarian sequences showed two clades supported by high posterior probabilities (PP) and bootstrap values (BV) (Fig 1). One monophyletic clade was represented by the family Thalassicollidae, and another clade was composed of three families, Collozoidae, Collophidae, and Collosphaeridae. The monophyletic Collosphaeridae clade was a sister to Collophidae, though three species of Collophidae were multidivergent. The clade consisting of Collophidae and Collosphaeridae was a sister to Collozoidae. This topology was almost the same as that inferred from maximum likelihood (ML) analysis.

In the clade consisting of Collophidae and Collosphaeridae, *Collophidium serpentinum* branched at the basal node. The clade Collosphaeridae nested within *Collophidium ellipsoides* and an environmental sequence AT8-54. In the clade Collosphaeridae, *Collosphaera globularis* and *Acrosphaera* sp. formed a monophyletic clade and branched together with *Siphonosphaera cyathina*.

The Collozoidae clade was composed of six species (*Collozoum inerme*, *C. pelagicum*, *Thalassophysa pelagica*, *Sphaerozoum ovodimare, Sphaerozoum punctatum*, and *C. amoeboides*) and an environmental sequence (IBEA.CTG.2022727) (Fig 1). *Collozoum inerme* formed a monophyletic clade with an environmental sequence IBEA.CTG.2022727 (moderate statistical support: 0.61 PP and 76% BV). The *Collozoum pelagicum* and *Thalassophysa pelagica* clade and the *Sphaerozoum ovodimare* and *S. punctatum* clade were also monophyletic. These three monophyletic clades formed a polyphyletic group and branched with *Collozoum amoeboides*. The phylogenetic relationships of the genus *Collozoum* were multidivergent.

The Thalassicollidae clade consisted of two species, *Thalassicolla pellucida* and *T. nucleate* (Fig 1). Three individual sequences of *T. nucleate* were monophyletic with 1.00 PP and 100% BV, though each of them was obtained from different geographic areas (North Pacific and North Atlantic). The *T. nucleate* clade branched with *T. pellucida*.

### Divergence time estimation

**Figure 2:**
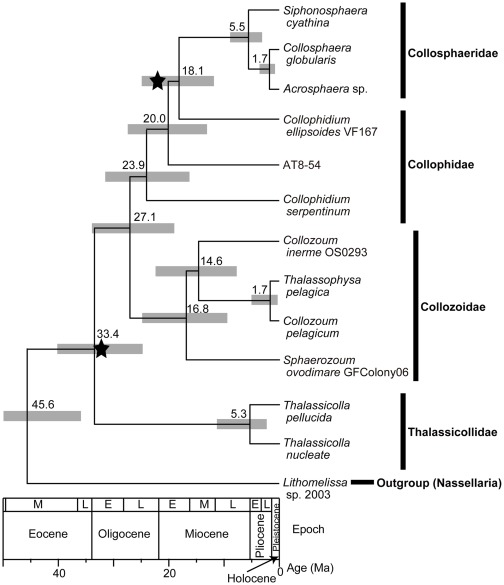
Divergence times among 12 collodarian sequences, based on the estimation of the partitioned Bayesian approach. Estimated divergence ages (million years ago) are shown at the root of each node. Gray shaded boxes on the nodes indicate 95% credible intervals. Star symbols represent the first appearance dates of Collosphaeridae and Collozoidae, as inferred from the fossil record [20], [21].

We estimated the divergence times of four families (Thalassicollidae, Collozoidae, Collophidae, and Collosphaeridae) identified as belonging to Collodaria (Fig 2). The common ancestor of Collodaria appeared around 45.6 Ma, and the credible interval (CI) was 35.9–49.9 Ma. The Thalassicollidae lineage and other collodarians diverged around 33.4 Ma ago (CI: 24.7–40.1 Ma). The divergence between the lineages of Collozoidae and a common ancestor of the clade consisting of Collophidae and Collosphaeridae was around 27.1 Ma ago (CI: 19.1–34.0 Ma), and that between Collophidae and Collosphaeridae was around 18.1 Ma ago (CI: 11.8–24.9 Ma).

## Discussion

### New taxonomic criteria for Collodaria inferred from molecular phylogeny

The molecular phylogeny of the SSU rDNA sequences formed four clades corresponding to classification at the family level (Fig 1). These families were clearly divided into two groups: (1) Thalassicollidae, and (2) Collozoidae, Collophidae, and Collosphaeridae. Notably, Thalassicollidae was the only collodarian family with a solitary lifestyle, whereas the other three families formed colonies (Table 1). Moreover, the cell structures of these two groups are consistent within each group. Thalassicollidae (solitary) have a hyaline gelatinous layer bounded by a central capsule and rhizopodia radiating outward [10], whereas the colonials have many interconnected cells linked inside by rhizopodia that protrude from the gelatinous envelope [11]. These morphological differences are consequently congruent with the molecular phylogenetic relationships between Thalassicollidae and the clade consisting of Collozoidae, Collophidae, and Collosphaeridae.

**Table 1:** Morphological features of collodarian species and family.

Family	Species	Life style	nucleus	Siliceous deposit	Central capsule
Collosphaeridae	*Collosphaera globularis*	Colony	**irregular**	**plate**	sphare
	*Acrosphaera* sp.	Colony	**?**	**plate**	sphare
	*Siphonosphaera cyathina*	Colony	**?**	**plate**	sphare
Collollophidium	*Collophidium ellipsoides*	Colony	**?**	None	**elongate**
	*Collophidium serpentinum*	Colony	**irregular**	None	**elongate**
Collozoidae	*Sphaerozoum punctatum*	Colony	sphere	**spicule**	sphare
	*Sphaerozoum ovodimare*	Colony	?	**spicule**	sphare
	*Collozoum inerme*	Colony	sphere	None	sphare
	*C. pelagicum*	Colony	?	None	**digitiform**
	*C. amoeboides*	Colony	?	None	**Amoeboid to lobate**
Thalassicollidae	*Thalassicolla nucleata*	**Solitary**	sphere	None	sphare
	*T. pellucida*	**Solitary**	?	None	sphare

Bold characters are showing characteristic morphologies.

Among the colonial collodarians, our molecular phylogeny separated the family Collozoidae from two others (Collophidae and Collosphaeridae), a separation consistent with morphological differences in the shapes of nuclei (Table 1). Collozoidae have spherical nuclei [11], whereas Collophidae and Collosphaeridae possess irregularly shaped nuclei (Collophidae [7]; Collosphaeridae [19]). On the basis of morphological taxonomy, the genus *Collophidium* has been assigned to the family Collozoidae, though the fact that this species has an irregularly shaped nucleus is different from the other Collozoidae [7]. Our phylogeny suggests that the genus *Collophidium* is independent from Collozoidae, a conclusion that supports attributing taxonomic significance to the shape of the nucleus. On the other hand, the genus *Collophidium* is also morphologically different from Collosphaeridae, because it has no siliceous skeleton, and the shape of the *Collophidium* central capsule is elongated (Table 1). The integration of molecular and morphological information thus argues that the genus *Collophidium* be separated from both Collosphaeridae and Collozoidae. We hence propose that the genus *Collophidium* be elevated to family status (Collophidae).

Molecular phylogeny has thus helped to clarify the morphological phylogenetic relationships among four collodarian families: Thalassicollidae, Collozoidae, Collophidae, and Collosphaeridae. The phylogenetic relationships initially divide these families into two groups, Thalassicollidae (solitary) and the others (colonial). Among the colonial groups, the shape of the nucleus is a key to distinguishing two sub-groups: (1) Collozoidae, and (2) Collophidae and Collosphaeridae. Collosphaeridae and Collophidae can be distinguished by the presence or absence of siliceous shells (Table 1). Use of such a new taxonomic scheme could help advance understanding of collodarian phylogeny and evolution.

### Evolutionary pattern of Collodaria

Our estimated divergence time of the common ancestor of Collodaria (Fig 2.; the middle Eocene) is much older than the oldest fossil record of collodarians (the Oligocene). The first appearance dates (FADs) of silicified Collozoidae and Collosphaeridae (32 and 22 Ma ago, respectively [20], [21]), although based on only a few fossil records, are almost consistent with our estimation. A paucity of taxonomic sampling has sometimes caused an inconsistency between molecular clock estimates and fossil records [22]. The divergence time of Collosphaeridae was slightly underestimated relative to the FAD of the fossils, probably because two of five genera in this family were excluded in the present study. However, the 95% credible interval at the Collosphaeridae node included the FAD of its fossils. Therefore, our estimated divergence times help to inform examination of the pattern of collodarian evolution throughout geological history.

The large gap between our estimation of the collodarian origin and the fossil record suggests that most ancestral collodarians lacked siliceous shells. Indeed, there are no siliceous deposits associated with most collodarian species, and such deposits are completely lacking in the family Thalassicollidae, which is directly descendant from the most ancestral lineage of Collodaria. The absence of a siliceous shell could have been one survival strategy in the first collodarian evolution, because some descendant families of Collodaria form siliceous shells.

**Figure 3:**
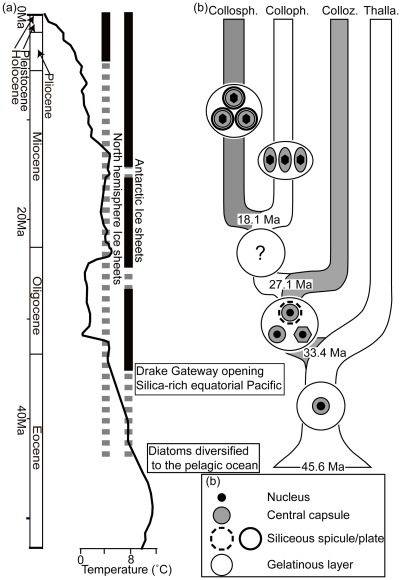
Schematic evolutionary model of Collodaria compared in relation with paleoceanographic environmental changes. (a) The black line shows the change of paleotemperature calculated from the stable oxygen isotopic (∂^18^O) values of the benthic foraminifera from the Eocence to recent [50]. The vertical bars indicate the presence of ice sheets and icebergs in each hemisphere, with the solid bar representing permanent presence of ice-sheets and the dash bar representing ephemeral presences [50], [51]. Two key geological events associated with the discussion are enclosed in the boxes [25]–[28], [31]–[34]. (b) Schematic evolutionary model of Collodaria and their divergence times. The estimated divergence time were shown on the nodes. Pictures on the tree represent schematic models of each collodarian cell characteristic. Abbreviations: Colloz, Collozoidae; Colloph, Collophidae; Collosph, Collosphaeridae; Thalla, Thalassicollidae. Each schematic cell structure is shown on the evolutionary tree.

We estimated the first divergence of the most ancestral collodarians (45.6 Ma ago) to have occurred in the middle Eocene (Fig 3). Since the middle Eocene the shell weights of other radiolarian fossils have been gradually decreasing [23]. Another study inferred that the extent of radiolarian skeletal silicification has decreased since the middle Eocene [24]. Contrarily, another silica consumer, diatoms, extended the range of their geographic distribution from the late Cretaceous and have spread into the pelagic ocean with large biomass since then [25]–[28]. Under these conditions the ancestral collodarians probably lost or suppressed their capacity to deposit silica owing to competitive selection pressures from other silica-secreting organisms.

We estimated the divergence of the first colonial collodarians to have occurred ca. 33.4 Ma ago in the early Oligocene (Fig 2). This date is consistent with the FAD of silicified Collozoidae, which are directly descended from the common ancestor of colonial collodarians in our phylogeny. Our data indicate that those collodarian ancestors became colonial approximately in the early Oligocene.

During the early Oligocene, the Antarctic region was cooling, and major ice sheets began to develop on the Antarctica [29]. Accordingly, the sea level was low [30] and a combination of weathering and erosion increased the export of silicates from the exposed shelf into the oceans [31]. The influx of lithogenic materials changed the equatorial Pacific to a silica-rich ocean. Diatoms were highly diverse during this period [32]; however, they did not become dominant in equatorial Pacific sediments until 17–15 Ma ago in the Miocene [33]. Therefore, competition with diatoms probably did not constrain reactivation of the ability of collodarians to silicify in the silica-rich equatorial Pacific during the early Oligocene.

During the same period of time, the Drake Passage opened [34]. This gateway allowed the establishment of the Antarctic Circumpolar Current, which promotes the thermal isolation of the Antarctica [35], and the development of deep bottom currents into the Pacific [36]. The present oceanic circulation system has been established since then [37]. Furthermore, the decrease in nutrient and productivity indicators (Ba, CaCO_3_) suggests that the equatorial and subtropical areas of the ocean became oligotrophic [38]. As a result of these oceanic environmental changes, the faunal composition of Radiolaria has drastically changed in the equatorial Pacific [39]. The first colonial collodarians could have evolved from ancestral collodarians (solitary) by acquiring a colonial lifestyle. The colonial lifestyle is one of the strategies to survive in the oligotrophic oceans because it enhances the availability of organic carbon supplied by highly productive symbionts [14], [16].

The occurrence of colonial collodarians could enable high primary productivity even in the oligotrophic subtropical and tropical oceans. Despite diminished nutrient supplies to tropical seas in the Oligocene, productivity was slightly higher than was the case during other low-productivity events in the Eocene [38]. The evolution of Collodaria may therefore have had an impact on primary productivity in the oligotrophic ocean.

## Materials and Methods

### Sampling

Samples to be analyzed were taken from four stations: one station in the North Pacific, two in the South Pacific, and one in the Mediterranean Sea with a North Pacific Standard Net (NORPAC) system (Table 2). After each net tow, we immediately isolated single cells or colonies from the samples and incubated them at 4°C for 2 to 6 hours to allow time for digestion of organisms that had been consumed by the Collodaria; the presence of these organisms would otherwise have contaminated the samples. After incubation, a photograph of each sample was taken (Fig. S2; there are no pictures for *Collozoum inerme* GFColony 3), and an isolated single cell of a colony, or a whole colony (see Table 2), was put into a 0.2-ml tube and kept at –80°C.

**Table 2:** Sample IDs and sampled locations.

Sample ID	Area	Long.	Lat.	Depth	Mesh size	Mesg type	Date
*Collozoum inerme* OS293	North Pacific	173°59'E	44°00'N	0–200m	100 µm	NORPAC	July 2007
*Collophidium ellipsoides* VF167	Mediterranean Sea	07°19'E	43°42'N	0–80m	100 µm	Open-closing net	July 2008
*Collozoum inerme* GFColony 3	South Pacific	161°12'E	4°30'S	0–200m	63 µm	Open-closing net	March 2008
*Sphaerozoum ovodimare* GFColony 6	South Pacific	160°01'E	8°09'S	0–200m	63 µm	Open-closing net	March 2008

**Table 3:** Sequences used for phylogenetic analysis.

Species name	Accession number
***Collozoum inerme*** ** OS0293**	**AB690555**
***Collozoum inerme*** ** GFColony03**	**AB690554**
*Collozoum inerme*	AY266295
*Thalassophysa pelagica*	AY266296
*Collozoum pelagicum*	AF091146
*Rhaphidozoum acuferum*	AF091147
*Sphaerozoum punctatum*	AB613246
***Sphaerozoum ovodimare*** ** GFColony06**	**AB690556**
*Collozoum amoeboides*	AB613245
*Collophidium serpentinum*	AF018162
***Collophidium ellipsoides*** ** VF167**	**AB690557**
*Collosphaera globularis-huxleyi*	AF018163
*Acrosphaera* sp. CR6A	AF091148
*Siphonosphaera cyathina*	AF091145
*Thalassicolla pellucida*	AY266297
*Thalassicolla nucleate*	AF018160
*Thalassicolla nucleate* W10.79	AF057744
*Thalassicolla nucleate* W10.74	AF057743
IBEA.CTG.2022727	2022727
AT8-54	AF530524
*Lithomelissa* sp. 8012	AB246694
*Lithomelissa* sp. 2003	AB246683
*Eucyrtidium hexagonatum*	AB179735
*Dictyocoryne profunda*	AB101540
*Larcopyle butschlii*	AB613231
*Sticholonche* sp.	AY268045
*Haliommatidium* sp.	AF018159
*Amphiacon denticulatus*	AB178585
*Phagomyxa odontellae*	AF310904
*Cercomonas longicauda*	AF411270

Bold species are from this study.

### Extraction and PCR

For DNA extraction, we crushed a cell in 50 µl of guanidine buffer (4 M guanidine isothiocyanate, 50 mM Tris [pH 7.4], 10 mM EDTA, 2% sarcosyl, 1% 2-mercaptoethanol) and incubated it at 70°C for 20 minutes. The supernatant was collected and used for polymerase chain reaction (PCR) amplification carried out with a denaturation step of 95°C for 5 minutes, followed by 35 amplification cycles at 95°C for 30 seconds, 56°C for 30 seconds, and 72°C for 2 minutes, and then a final extension at 72°C for 10 minutes. PCR amplification for the most complete SSU rDNA was amplified by two overlapping fragments of combinations of the following primers: universal forward primer SA (5′-AAC-CTG-GTT-GAT-CCT-GCC-AGT-3′) and newly designed reverse primer S81NC (5′-TCA-CAG-ACC-TGT-TAT-TGC-HW-3′ and newly designed forward primer S50NC (5′-GGA-AGG-GCA-CCA-CA-3′) and universal reverse primer SB (5′-TGA-TCC-TTC-TGC-AGG-TTC-ACC-TAC-3′). PCR products were ligated and cloned with a TOPO TA-Cloning Kit (Invitrogen, Carlsbad, CA, USA), and purified with an AMPure Kit (Beckman Coulter, Brea, CA, USA). We sequenced these samples with a Big Dye Terminator Cycle Sequencing Kit (Applied Biosystems, Carlsbad, CA, USA) and the ABI PRISM 3100 Genetic Analyzer (Applied Biosystems) at Station Biologique de Roscoff (France). From each PCR product, we sequenced more than 16 clones to confirm the absence of polymorphisms of the SSU rDNA in a single cell. A representative sequence was selected from the clones of four individuals, and four sequences excluding the regions of primers at the 5′ and 3′ ends of the SSU rDNA were deposited in the DDBJ DNA databank (accession numbers AB690554 to AB690557).

### Phylogenetic analysis

We aligned two datasets of the SSU rDNA sequences. The first dataset contained 19 collodarian sequences (four species obtained in this study, 13 from isolated samples, and two environmental collodarian sequences from other studies), eight other radiolarians, and two cercozoans as outgroups. The second dataset was composed of 19 collodarians and three nassellarian sequences as outgroups listed in Table 3. These two datasets were aligned by using CLUSTALX version 2.0 [40] and manually refined. We used 1241 and 1407 unambiguous nucleotide positions for phylogenetic analysis of the first and second datasets, respectively. The best-fit model of nucleotide substitution for these datasets was selected by using MrModeltest [41] and Treefinder [42]. Both maximum-likelihood (ML) and Bayesian analysis were performed under the general time reversible model [43] with the gamma distribution model (G [44]) for both datasets. Phylogenetic trees were created by using Bayesian analysis with MrBayes Version 3.1.2. [45]. The Markov chain Monte Carlo (MCMC) process was set so that four chains (three heated and one cold) ran simultaneously. Two independent runs were subsequently conducted; both runs were continued for 1.0×10^6^ cycles after stationarity had been reached. We confirmed agreement of the estimated parameters between the two independent runs. Then we pooled all trees from both runs after a burn-in period. Posterior probabilities were estimated from the pooled trees. The MLs of two datasets were performed by using Treefinder. Bootstrap support was based on 1000 replicates.

### Divergence time estimation

Analyses of divergence times were conducted with the program Thornian Time Traveler ver.1.0 (T3; see http://abacus.gene.ucl.ac.uk) in accord with the Bayesian method [46]. The analyses were applied to the 13 sequences *Collozoum inerme* OS293, *Collozoum pelagica*, *Thalassophysa pelagica*, *Sphaerozoum ovodimare* GF Colony6, *Collophidium serpentinum*, AT8-54, *Collophidium ellipsoides* VF167, *Collosphaera globularis*, *Acrosphaera* sp, *Siphonosphaera cyathina*, *Thalassicolla pellucida*, and *Thalassicolla nucleate*, with a final rooting based on *Lithomelissa* sp. 2003. We used the same dataset with phylogenetic reconstruction and obtained the tree topology for 14 sequences by using MrBayes3.1.2. Branch lengths of this topology were estimated with the estbNew program (T3). The F84 [47] + G model (the most parameter-rich model in T3) was used with parameters estimated by using the PAML ver. 3.14 package [48]. Then the divergence time was estimated by using the multidivtime program T3. The MCMC approximation was made within a burn-in period of 1.0×10^5^ proposal cycles. Samples of the Markov chain were taken every 100 cycles until a total of 10,000 samples were obtained. To diagnose possible failure of the Markov chains to converge to their stationary distribution, we performed two replicate MCMC runs with different initial starting points for each analysis. The multidivtime program requires a value for the mean of the prior distribution for the time separating the ingroup root from the present (rttm). We used 91 Ma ago for this estimation, on the basis of the first appearance of the genus *Lithomelissa* [49].

## Supporting Information

Figure S1 **Bayesian phylogeny of the SSU rDNA sequences obtained from the radiolarians.** Four novel collodarian sequences and two environmental sequences (<3 µm) are shown in bold and light gray text, respectively. Order names are shown to the right of the balck bars. Numbers on nodes indicate posterior probabilities of the Bayesian method and bootstrap values of the ML analysis. Scale bar located at lower left shows 0.1 substitutions per site for the Bayesian analyses.
(TIF)

Figure S2 **Photographs of specimens.** Black bar is 30 µm. (a) *Collozoum inerme* OS293, (b) *Collophidium ellipsoides* VF167, (c) *Sphaerozoum ovodimare* GFColony6.
(TIF)

## References

[1] Cavalier-Smith T, Chao EE (2003). Phylogeny and classification of phylum Cercozoa (Protozoa).. Protist.

[2] Anderson OR, Nigrini C, Boltovskoy D, Takahashi K, Swanberg NR, Lee JJ, Leedale GF, Bradbury P (2002). Class Polycystina.. The second illustrated guide to the Protozoa.

[3] Kunitomo Y, Sarashina I, Iijima M, Endo K, Sashida K (2006). Molecular phylogeny of acantharian and polycystine radiolarians based on ribosomal DNA sequences, and some comparisons with data from the fossil record.. Eur J Protistol.

[4] Strelkov AA, Reshetnyak VV (1971). Colonial spumellarian radiolarians of the world ocean.. In Academy of Sciences, USSR, Explorations of the fauna of the seas.

[5] Haeckel E (1862). Die Radiolarien (Rhizopoda Radiaria): Eine Monographie.

[6] Haeckel E (1887). Report on the Radiolaria collected by H. M. S. Challenger during the years 1873–1876..

[7] Anderson OR, Gastrich MD, Zettler LA (1999). Fine structure of the colonial radiolarian *Collozoum serpentinum* (Polycystinea: Spumellaria) with a reconsideration of its taxonomic status and re-establishment of the genus *Collophidium* (Haeckel).. Mar Micropaleontol.

[8] De Wever P, Dumitrica P, Caulet JP, Nigrini C, Caridroit M (2001). Radiolarians in the sedimentary record..

[9] Ishitani Y, Ujiié Y, De Vargas C, Not F, Takahashi K (2012). Two distinct lineages in the radiolarian Order Spumellaria having different ecological niches.. Deep-Sea Res Pt II.

[10] Anderson OR (1976a). A cytoplasmic fine-structure study of two spumellarian Radiolaria and their symbionts.. Mar Micropaleontol.

[11] Anderson OR (1976b). Ultrastructure of a collodarian radiolarian (*Sphaerozoum punctatum* Müller 1858) and cytochemical determination of the role of its zooxanthellae.. Tissue Cell.

[12] Polet S, Berney C, Fahrni J, Pawlowski J (2004). Small-subunit ribosomal RNA gene sequences of Phaeodarea challenge the monophyly of Haeckel's Radiolaria.. Protist.

[13] Stoecker DK, Hohnson MD, De Vargas C, Not F (2009). Acquired phototrophy in aquatic protists.. Aquat Microb Ecol.

[14] Anderson OR, Levandowsky M, Hunter SH (1980). Radiolaria. Biochemistry and physiology of Protozoa.

[15] Swanberg NR, Harbison GR (1980). The ecology of *Collozoum longiforme*, sp. nov., a new colonial radiolarian from the equatorial Atlantic Ocean.. Deep-Sea Res.

[16] Anderson OR, Swanberg NR, Bennett PB (1983). Assimilation of symbiont-derived photosynthates in some solitary and colonial radiolaria.. Mar Biol.

[17] Khmeleva NN (1967). Role of the radiolarians in the value of primary productivity in the Red Sea and Gulf of Aden.. Doklady Akademii Nauk SSSR.

[18] Taylor FJR, Capriulo GM (1990). Symbiosis in marine protozoa.. The ecology of marine protozoa.

[19] Anderson OR (1978). Fine structure of a symbiont-bearing colonial radiolarian, *Collosphaera globularis* and ^14^C isotopic evidence for assimilation of organic substances from its zooxanthellae.. J Ultra Struct R.

[20] Bjørklund KR, Goll RM (1979). Internal skeletal structures of Collosphaera and Trisolenia: a case of repetitive evolution in the Collosphaeridae (Radiolaria).. J Paleontol.

[21] Riedel WR, Harland WB (1967). Subclass Radiolaria.. The fossil record.

[22] Hugall AF, Lee MS (2007). The likelihood node density effect and consequence for evolutionary studies of molecular rates.. Evolution.

[23] Moore TC (1969). Radiolaria: change in skeletal weight and resistance to solution.. Geological Society of America Bulletin.

[24] Lazarus DB, Kotrc B, Wulf G, Schmidt DN (2009). Radiolarians decreased silicification as an evolutionary response to reduced Cenozoic ocean silica availability.. Proc Natl Acad Sci U S A.

[25] Fenner J (1985). Bolli HM, Saunders JB, Perch-Nielsen K, editors..

[26] Maliva RG, Knoll AH, Siever R (1989). Secular change in chert distribution: A reflection of evolving biological participation in the silica cycle.. Palaios.

[27] Harwood DM, Nikolaev VA (1995). Blome CD, Whalen PM, Reed KM, editors..

[28] Nikolaev VA, Harwood DM (2000). Economou-Amilli A, editor..

[29] Shevenell AE, Kennet JP (2007). Cenozoic Antarctic cryosphere evolution: Tales from deep-sea sedimentary records.. Deep-Sea Res Pt II.

[30] Miller KG, Browning JV, Aubry MP, Wade BS, Katz ME (2008). Eocene-Oligocene global climate and sea-level changes: St. Stephens Quarry, Alabama.. Geol Soc Am Bull.

[31] Merico A, Tyrrell T, Wilson PA (2008). Eocene/Oligocene ocean de-acidification linked to Antarctic glaciation by sea-level fall.. Nature.

[32] Rabosky DL, Sorhannus U (2009). Diversity dynamics of marine planktonic diatoms across the Cenozoic.. Nature.

[33] Barron JA, Baldauf JG, Berger WH, Smetacek VS, Wefer G (1989). Tertiary cooling steps and paleoproductivity as reflected by diatoms and biosiliceous sediments.. Productivity of the ocean: Present and past.

[34] Diekmann B, Kuhn G, Gersonde R, Mackensen A (2004). Middle Eocene to early Miocene environmental changes in the sub-Antarctic Southern Ocean: evidence from biogenic and terrigenous depositional patterns at ODP site 1090.. Global Planet Change.

[35] Kennett JP (1977). Cenozoic evolution of Antarctic glaciation, the circum-Antarctic Ocean, and their impact on global paleoceanography, J Geophys Res.

[36] Carter L, Carter RM, McCave IN (2004). Evolution of the sedimentary system beneath the deep Pacific inflow off eastern New Zealand.. Mar Geol.

[37] Huber M, Caballero R (2003). Eocene El Nino: Evidence for robust tropical dynamics in the “hothouse”.. Science.

[38] Griffith E, Calhoun M, Thomas E, Averyt K, Erhardt A (2010). Export productivity and carbonate accumulation in the Pacific Basin at the transition from a greenhouse to icehouse climate (late Eocene to early Oligocene).. Paleoceanography.

[39] Funakawa S, Nishi H, Moore T, Nigrini CA (2006). Radiolarian faunal turnover and paleoceanographic change around Eocene/Oligocene boundary in the central equatorial Pacific, ODP Leg 199, Holes 1218A, 1219A, and 1220A.. Palaeogeogr, Palaeoclimatol, Palaeoecol.

[40] Thompson JD, Gibson TJ, Plewniak F, Jeanmougin F, Higgins DG (1997). The CLUSTAL_X windows interface: flexible strategies for multiple sequence alignment aided by quality analysis tools.. Nucleic Acids Res.,.

[41] Nylander JAA (2004). MrModeltest v2..

[42] Jobb G (2008). http://www.treefinder.de.

[43] Tavaré S (1986). Some probabilistic and statistical problems on the analysis of DNA sequence.. Lecture of Mathematics for Life Science.

[44] Yang Z (1994). Maximum likelihood phylogenetic estimation from DNA sequences with variable rates over sites: approximate method.. J Mol Evol.

[45] Ronquist F, Huelsenbeck JP (2003). MRBAYES 3: Bayesian phylogenetic inference under mixed models.. Bioinformatics 19,.

[46] Thorne JL, Kishino H (2002). Divergence time and evolutionary rate estimation with multilocus data.. Syst Biol.

[47] Felsenstein J (1984). Distance methods for inferring phylogenies: a justification.. Evolution.

[48] Yang Z (1997). PAML: a program package for phylogenetic analysis by maximum likelihood.. CABIOS.

[49] Cault JP (1991). Radiolarians from the Kerguelen Plateau, Leg 119.. Proceedings of the Ocean Drilling Program, Scientific results.

[50] Zachos JC, Pagani M, Sloan L, Thomas E, Billups K (2001). Trends, rhythms, and aberrations in global climate 65 Ma to present.. Science.

[51] Moran K, Backman J, Brinkhuis H, Clemens SC, Cronin T (2006). The Cenozoic paleaeoenvironment of the Arctic Ocean.. Nature.

