# Supplementary figures and images for: Phylogenetic Relationships and Evolutionary Patterns of the Order Collodaria (Radiolaria)
Source: PLoS One. 2012 May 2;7(5):e35775. doi: 10.1371/journal.pone.0035775 (PMC3342292; doi:10.1371/journal.pone.0035775)

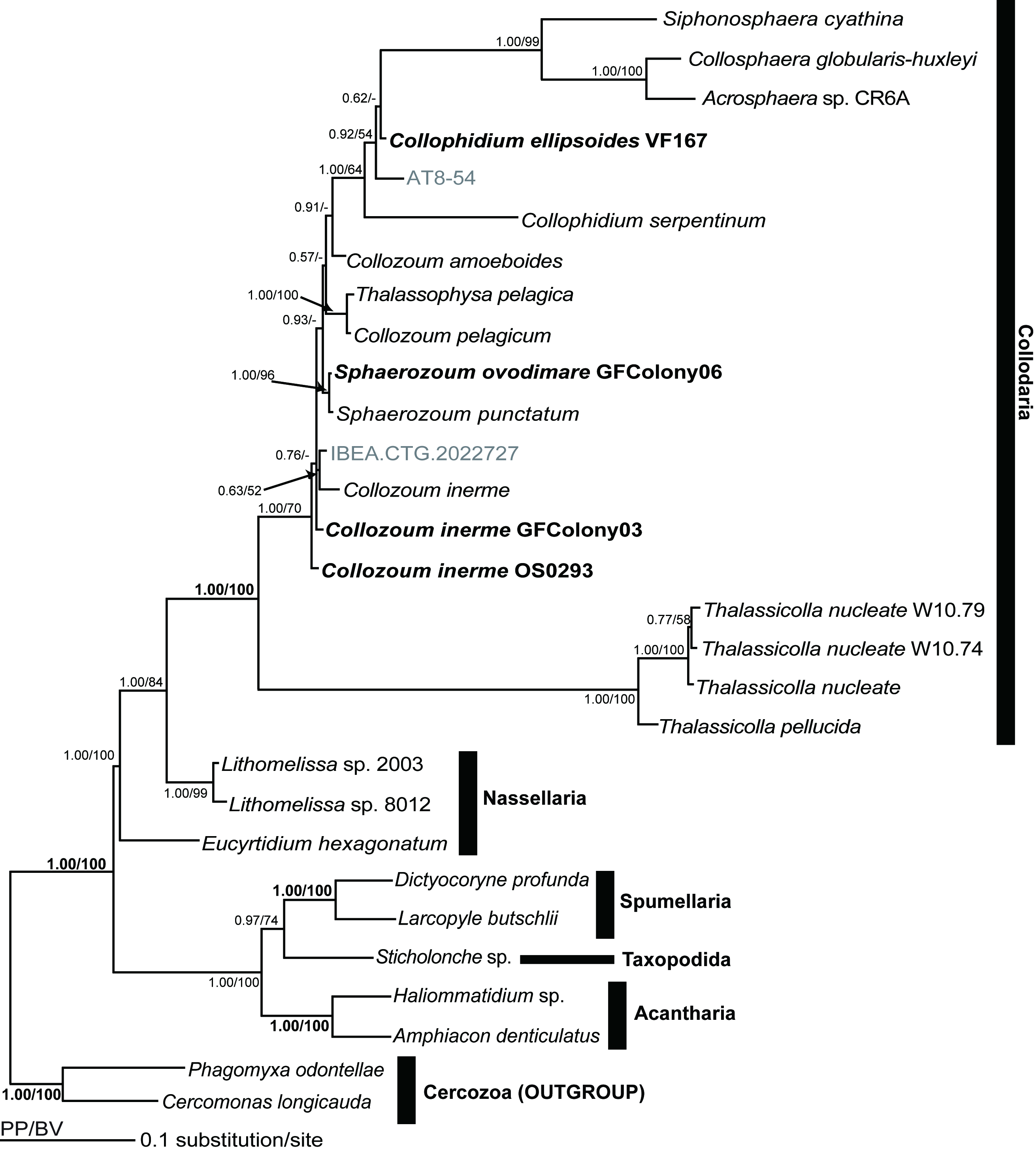

Supplement: Figure S1 — Bayesian phylogeny of the SSU rDNA sequences obtained from the radiolarians. Four novel collodarian sequences and two environmental sequences (<3 µm) are shown in bold and light gray text, respectively. Order names are shown to the right of the balck bars. Numbers on nodes indicate posterior probabilities of the Bayesian method and bootstrap values of the ML analysis. Scale bar located at lower left shows 0.1 substitutions per site for the Bayesian analyses. (TIF) [file pone.0035775.s001.tif]

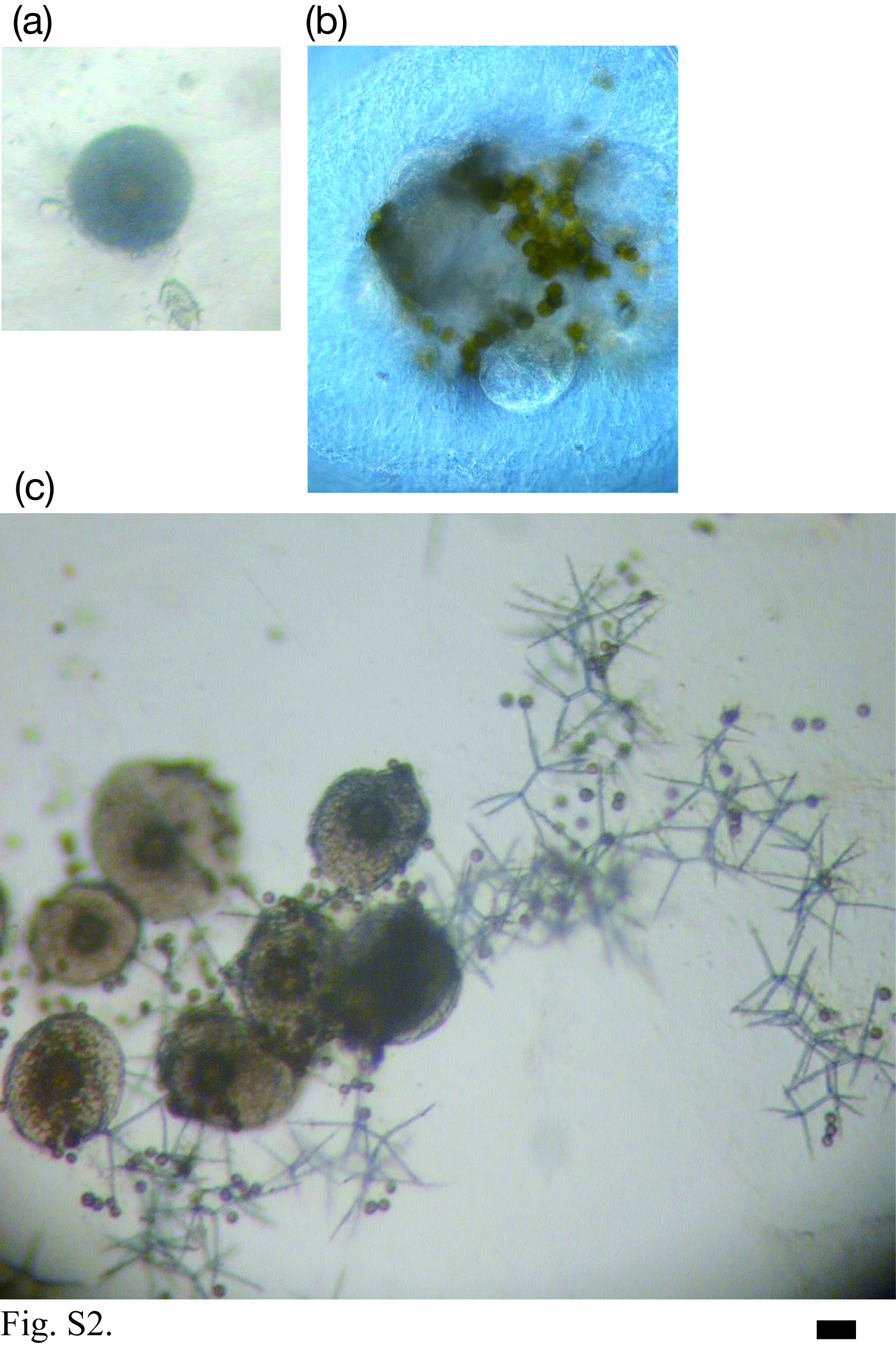

Supplement: Figure S2 — Photographs of specimens. Black bar is 30 µm. (a) Collozoum inerme OS293, (b) Collophidium ellipsoides VF167, (c) Sphaerozoum ovodimare GFColony6. (TIF) [file pone.0035775.s002.tif]
